# Inequalities in Birth Weight in Relation to Maternal Factors: A Population-Based Study of 3,813,757 Live Births

**DOI:** 10.3390/ijerph19031384

**Published:** 2022-01-26

**Authors:** Agnieszka Genowska, Radosław Motkowski, Vaiva Strukcinskaite, Paweł Abramowicz, Jerzy Konstantynowicz

**Affiliations:** 1 Department of Public Health, Medical University of Bialystok, 15-295 Bialystok, Poland; 2 Department of Pediatrics, Rheumatology, Immunology and Metabolic Bone Diseases, Medical University of Bialystok, University Children’s Hospital, 15-274 Bialystok, Poland; radek@umb.edu.pl (R.M.); pawel.abramowicz@umb.edu.pl (P.A.); jurekonstant@o2.pl (J.K.); 3 Faculty of Health Care, Vilnius University of Applied Sciences, LT-08303 Vilnius, Lithuania; vaiva.struk@gmail.com

**Keywords:** weight at birth, Z-score, newborn, advanced maternal age (AMA), parity, educational level, Poland

## Abstract

Background: Despite numerous studies of women having children later in life, evidence of the relationship between maternal factors and newborn outcomes in Central and Eastern European countries is limited. This study aimed to examine the association between maternal age, biological determinants, including parity and sex of the newborn, demographic and social background, and birth weight in 3.8 million singleton live births in Poland. Methods: The effect of maternal age on birth weight (in grams and Z-scores) adjusted for confounders was assessed using Generalized Linear Models. Results: The mean (±SD) birth weights of neonates born to primiparous women and multiparous women were 3356.3 ± 524.9 g and 3422.7 ± 538.6 g, respectively, which corresponded to a Z-score of −0.07 ± 0.96 and 0.14 ± 1.00, respectively (*p* ≤ 0.001). After controlling for biological, demographic, and social factors, a significant decrease in birth weight was found for primiparous women of the age group ≥30 years and multiparous women aged ≥35 years compared to the age group of 25–29 years. The lowest neonatal birth weight was observed in the case of women aged ≥45 years. Confounders did not affect birth weight Z-scores among primiparous women, whereas among multiparous women, together with educational factors, they reversed Z-scores from positive to negative values. The lower birth weight of neonates was overall associated with lower maternal education. Conclusions: Regardless of parity, advanced maternal age is strongly associated with a decreased neonatal birth weight, implying complications in early pregnancy and the antenatal period as well as obstetric complications. Counseling to support women’s family planning decisions and improving women’s education during their reproductive age may help to alleviate unfavorable newborn outcomes.

## 1. Introduction

Birth weight is a critical parameter for assessing neonatal health and a predictor of newborn growth and survival [1]. Neonatal traits or anthropometric measures are essentially determined by gestational age, the course of pregnancy, and other associated factors, such as pre- and perinatal factors. Although genetic determinant and biological effects in utero (fetal, placental, and maternal) are the major factors influencing the antenatal development and final neonatal size, some other important predictors of birth weight, such as age, parity, and maternal anthropometry have also been reported to play a role [2,3,4]. Furthermore, factors such as social status-related support, lifestyle (i.e., diet and smoking), and health care may explain birth weight inequalities among neonates [5].

Studies on newborn health conducted in developed countries [6,7,8] and in low- and middle-income countries [2] have suggested an inverse U-shaped relationship between maternal age and birth weight. However, data on these relationships in Central and Eastern European countries are limited [9,10]. In countries such as Poland, where significant changes or second demographic transition coincide with political reforms during the last decades [11,12], the identification of birth weight-related factors can be interesting. The demographic crisis worsened as a result of reforms that improved education, widened access to higher education, and increased the professional activity of women. All these processes eventually led to a shift in first-time childbearing age from 25.8 years old (recorded in 1990) to 30.3 years old (2019), and the percentage of live births associated with advanced maternal age (AMA, i.e., >34 years of age) nearly doubled from 8.9% to 19.5% [11,13,14]. Postponing or delaying births to a later age has been linked with biological restrictions in older mothers and an increased risk of obstetric complications, chromosomal abnormality, birth defects, miscarriages, or stillbirths [4,15,16,17]. As a consequence, the dynamic changes that occurred in Poland contributed not only to a sharp decrease of fertility to a point where generational replacement may have been lower than expected but also to significant differences in newborn outcomes.

So far, studies have not focused much on explaining the causes of differences in birth weight in Poland, which is the largest country in Central and Eastern Europe, with an annual number of live births of around 400,000. Research on the Polish population has analyzed the association of respiratory distress syndrome in preterm infants with social and demographic factors of mothers [18] and changes in birth weight depending on parity and mothers’ and fathers’ occupation [19]. Among the socioeconomic determinants, unemployment has been identified as the factor explaining most of the differences in birth weight [20]. However, the influence of maternal characteristics on newborn outcomes has not been empirically investigated in this large population.

The aim of this study was to examine the associations between maternal characteristics, such as age, biological determinants (gestational age, obstetric history, parity), demographic and social background (marital status, place of residence, education level), and birth weight in 3.8 million singleton live births in Poland, using data collected from the Polish Birth Registry.

## 2. Materials and Methods

In this retrospective study, we used the data collected from the Central Statistical Office (CSO) in Poland on live births based on 3,918,344 individual records. Cases of live births registered from 2008 to 2017 were included in the study. Because the reports are based on birth certificates and the Polish law requires each birth to be registered, the records covered every birth in the analyzed decade. Data of live births were transferred from obstetric and newborn medical records in each hospital using a standardized document for the entire country [13]. Individual birth certificate records were linked to the mother’s social variable (marital status) from certificates stored electronically for the years 2008–2017.

### 2.1. Newborn Outcomes

The primary outcome of interest was birth weight, which was recorded in grams and measured directly at birth. Birth weight converted into Z-scores was calculated separately for subgroups defined by sex and gestational age (with 1-week precision) by subtracting the group using standard deviations [21]. To determine Z-score, the following standard formula was used: Z-score = (birth weight—SD for gestational age and sex)/median for gestational age and sex [22]. These Z-scores describe the variations in birth weight regardless of gestational age and sex of newborns, allowing to compare boys with girls and more mature children with those less mature. For normally distributed variables, the 50th percentile was equal to median and mean (value 0), while Z-scores of −2 and −1 corresponded to 2.3 and 15.9 percentiles, and values of +1 and +2 corresponded to 84.1 and 97.7 percentiles, respectively. The sex of the newborns was recorded as boy or girl.

### 2.2. Maternal Characteristics

Maternal age was stratified into the following age groups: 20–24, 25–29, 30–34, 35–39, 40–44, and ≥45 years, with the range of 25–29 years considered as reference. Based on the number of previous live births, parity was stratified into primiparity and multiparity. The biological variable of the mothers was gestational age, which was defined as preterm (<37 full weeks), term (37–41 full weeks), and post-term (≥42 full weeks). Stillbirth in any previous gestation was defined as fetal death after the 22nd week of gestation [13].

Previous studies focusing on maternal age and birth weight indicated that demographic and social factors had a potential impact on newborn outcomes [16,23,24]. Demographic factors included marital status (married and unmarried) and place of residence (urban and rural area), while social factors were the level of education, which was classified based on the International Standard Classification of Education (ISCED) as low (categories 1–2), medium (categories 3–4), and high (categories 5–8) [25]. All these potential confounding factors were included in the multivariate model of the study.

**Figure 1:**
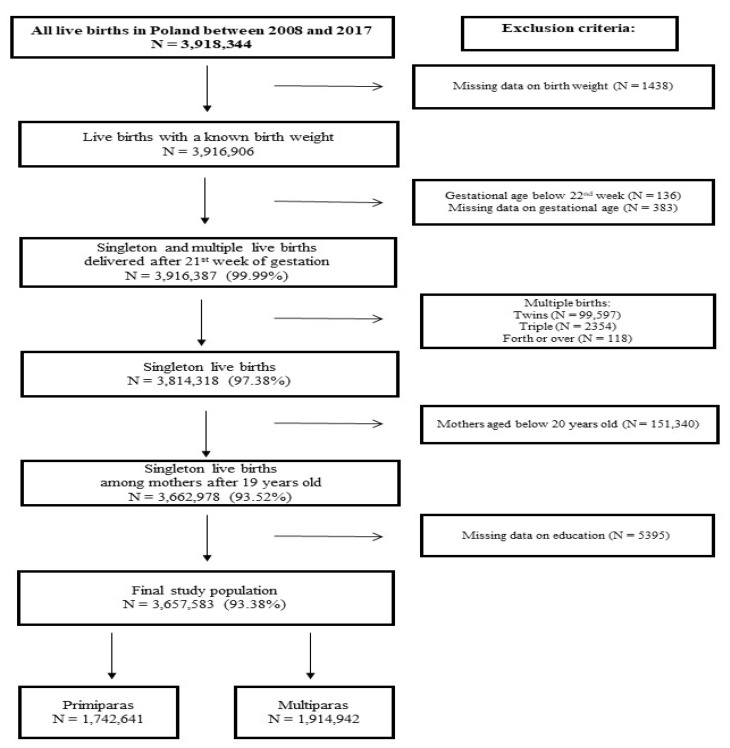
Flow diagram of the study.

The potential individual mediating factors included maternal characteristics, which were obtained from the data available from the Polish Central Statistical Office and were used as confounding factors as appropriate. We assumed that multiple births may significantly influence the association between maternal age and birth weight [26] and that mothers of young age (<20 years old) are heterogeneous and often a burdened group [27,28]; therefore, these records were excluded from the study (Figure 1). 

### 2.3. Statistical Analysis

To analyze the relationships between newborn outcomes (newborn weight by sex) and maternal factors, Generalized Linear Models with continuous dependent variables were used (assuming a normal distribution of model errors, which were transformed using the identity link function). Covariance matrices of the models were determined using model-based estimators (the negative of the generalized inverse of the Hessian matrix). The influence of maternal age on the birth weight of neonates was estimated as a linear and quadratic trend.

Results for birth weight were presented as the coefficients (β) of the regression models with 95% confidence intervals (CIs), which should be interpreted as estimated differences in birth weight in grams between particular maternal age category and reference age category. Regression models for birth weight Z-scores, including maternal age groups, were presented using expected values of the dependent variable as birth weight Z-scores with 95% confidence intervals (CIs). Independent factors were identified and analyzed by univariate regression, and multivariate regression analyses, taking into account the possible confounding factors. Four models were constructed to investigate the associations between maternal variables and birth weight by sex of newborns: model 1 included only maternal age; model 2 was adjusted for biological factors (gestational age and stillbirth history); model 3 included those variables analyzed in model 2 as well as demographic factors (marital status and place of residence); and model 4 additionally included education, an important social factor. All the analyses were conducted including stratification for parity and sex of neonates according to the approach reported elsewhere [3,7,29,30].

Statistical hypotheses were verified at a significance level of 0.05. All calculations were performed in IBM^®^ SPSS^®^ Statistics for Windows, Version 26.0 (IBM Corp., Armonk, NY, USA).

## 3. Results

During 2008–2017, a total of 3,657,583 singleton live births were recorded, of which 1,883,367 were boys (51.5%). A total of 1,742,641 births (47.6%) were recorded among primiparous women. The maternal and newborn characteristics analyzed in the study are presented in Table 1. The mean (± SD) maternal age of primiparous and multiparous women was 27.2 ± 4.3 and 30.8 ± 4.7 years, respectively.

The mean birth weight of neonates born to primiparous women was 3356.3 ± 524.9 g, which was lower by 66.4 g compared to neonates born to multiparous women (3422.7 ± 538.6 g, *p* ≤ 0.001). A stepwise decrease of birth weight was observed in the case of neonates born to primiparous women aged over 25–29 years and in multiparous women aged above 30–34 years. The lowest birth weight of neonates was observed for mothers ≥ 45 years of age (primiparous women: 3106.9 ± 627.0 g, multiparous women: 3258.3 ± 663.8 g, *p* ≤ 0.001). The results differed significantly by sex of newborns (*p* ≤ 0.001), with boys having a higher birth weight than girls (primiparous women: 3419.8 ± 535.6 g vs. 3288.8 ± 504.6 g, multiparous women: 3491.1 ± 548.8 g vs. 3350.2 ± 517.9 g) (Table 2).

For primiparous women, the mean and median birth weight in Z-score for each maternal age group was less than 0 (total: −0.07 ± 0.96). On the other hand, for multiparous women, the total Z-score had a positive value (0.14 ± 1.00), except in the case of the age group 20–24 years, for which the Z-score was found to be negative (−0.02 ± 1.00). A similar trend was found for boys and girls, i.e., negative Z-scores for primiparous women and positive values for multiparous ones, except for those aged 20–24 years. The highest mean and median Z-scores were found for primiparous mothers aged 25–29 years old (−0.05 ± 0.95) and multiparous mothers aged 30–34 years old (0.18 ± 0.99). A significant difference in Z-scores between boys and girls was found for mothers in the age groups 20–24, 25–29, and 30–34 years (*p* ≤ 0.001), but in mothers above 34 years of age, the differences were insignificant (Table 2).

Differences in birth weight between maternal age groups stratified by parity and neonate sex were statistically significant in the crude model and after controlling for biological, demographic, and social factors. All models showed that the relationships were linear and also a nonlinear inverse U-shape, while model 4 showed a weak quadratic relationship for the birth weight of boys born to primiparous women. When models 1–3 were applied for primiparous women, the differences in birth weight (β) were negative for both sexes and all mother age groups, compared with the reference age group 25–29 years (*p* ≤ 0.001). The inclusion of educational level in model 4 led to a significant increase in the difference in birth weight among primiparous women aged 20–24 years in comparison to the referenced age group. For primiparous women aged 29 years and older, the negative difference in birth weight (β) continued to systematically increase, and for mothers with most advanced age (≥45 years), the significant difference in β was −131.0 in boys, and −192.3 g in girls. For multiparous women, models 1–3 showed a distinct positive difference in the birth weight of girls born to those aged 30–34 years vs. the reference group. Significant positive differences in the birth weight of boys were found for the same maternal age in models 2 and 3. A continuous decrease in newborn birth weight was observed for multiparous women above 34 years of age. After adjustment for confounders, in those aged ≥45 years, the significant decrease of β value in boys and girls was −99.4 and −101.9 g, respectively (Table 3).

The results of the models including birth weight Z-score showed explicitly negative Z-scores for boys and girls born to primiparous women in all age groups, even after adjusting for biological, demographic, and social confounders. Birth weight Z-scores were positive for all multiparous women, except for the age groups 20–24 years and over 45 years, and the results persisted even after considering the biological and demographic factors. However, the inclusion of education (social factor) led to a shift of birth weight Z-scores from positive to negative for the majority of the multiparous women. Regardless of parity, the educational factor was strongly associated with a decreased Z-score for the birth weight compared with other confounders (Table 4).

**Figure 2:**
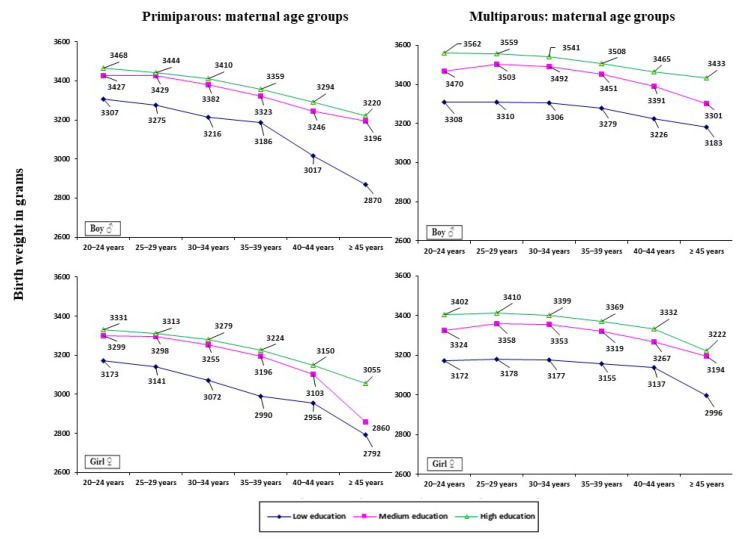
Birth weight based on the level of mother’s education (according to ISCED) for maternal age groups stratified by parity and sex of neonates.

Figure 2 demonstrates the impact of a mother’s education (low, medium, high) on birth weight, stratified by maternal age and parity. Mothers with a low level of education gave birth to neonates with the lowest birth weight, whereas those with a high educational level delivered neonates with the highest birth weight (*p* ≤ 0.001). Furthermore, the differences in birth weight were found to be more pronounced among multiparous women compared to primiparous ones. Among primiparous women, the difference in newborn birth weight between mothers with low vs. high levels of education was significant, and was on average −140.5 g in boys and −144.3 g in girls, whereas among multiparous women it was on average −236.1 and −223.0 g, respectively. Smaller differences in newborn birth weight were found between mothers with a low vs. medium level of education (primiparous women: −127.3 g in boys and −134.1 g in girls, multiparous women: −181.7 and −171.1 g, respectively). The smallest differences in newborn birth weight were found between mothers with a medium vs. high level of education (primiparous women: −13.3 g in boys and −10.2 g in girls, multiparous women: −54.4 and −51.9 g, respectively).

## 4. Discussion

Our study showed that AMA had a significant impact on newborn birth weight. A stepwise decrease of birth weight was observed for primiparous women from the age group 25–29 years to the age group ≥45 years (from 3373.1 to 3106.9 g), and for multiparous women who were older than 30–34 years (from 3441.5 to 3258.3 g, respectively). The associations between maternal age and birth weight were linear and also an inverse U-shaped curve and persisted even after adjusting for biological, demographic, and social characteristics of the mothers. In the multivariate model including the educational factor, an increasing trend of newborn birth weight was observed only for primiparous mothers in the age group 20–24 years. The estimated birth weight Z-scores were negative for primiparous women. Confounding factors did not change the results. However, in the case of multiparous women, the Z-score was generally positive, but after adjusting for education the values changed to negative. Parity was a factor associated with birth weight; for primiparous women, the mean birth weight for each maternal age group was significantly lower in comparison to multiparous women. Additionally, differences in birth weight were found in relation to the sex of the neonates: the weight at birth was about 4% greater in boys than in girls.

The associations between maternal age and birth weight were found to be highly specific for each maternal age group—linear and nonlinear (i.e., inverse U-shaped). The findings were observed to be similar for multiparous and primiparous mothers, both in unadjusted analyses and after adjustment for confounders. Newborns of the AMA group had a significantly lower birth weight compared to those of mothers of the age group 20–24 years. These results are in line with those reported by studies conducted in high-, middle-, and low-income countries, despite the use of different estimation methods [2,6,7,8].

Although we could not identify the mechanisms underlying the observed associations, some explanations can be given. For example, in the case of the AMA group, biological conditions, particularly those associated with the reproductive system, i.e., the body aging processes, are associated with stepwise functional involution, accelerated placental aging, and detrimental changes in nutrient transport and vascular functions. In addition, AMA is associated with an increased risk of chronic diseases, including hypertension and diabetes, which usually result in impaired placental function and in utero undernutrition of the fetus [4,8,31].

Adverse newborn outcomes such as lower birth weight (commonly observed among older mothers) are associated with both short- and long-term consequences, including neonatal health problems in later life. Low birth weight has been identified as a predictor of mortality among preterm newborns, owing to an increased risk of bronchopulmonary dysplasia and necrotizing enterocolitis [1,32]. It has also been linked to the development of hypertension, coronary heart diseases, type 2 diabetes, and chronic kidney diseases in the later life of offspring. In utero programming of chronic disorders is a self-propelling mechanism of a vicious circle associated with several diseases in future generations [33,34,35].

Importantly, the results of our study also showed that parity was strongly associated with birth weight, while a greater newborn birth weight observed in the case of multiparous mothers had a rather biological etiology. It seems that the volume of the uterus increases following the first pregnancy, along with an increase in the capacity of uterine and placental blood flow, which subsequently leads to better fetal growth [36]. In the present study, both primiparous and multiparous mothers in the advanced age groups delivered smaller neonates in relation to mothers in the age group 20–24 years. These observations suggest that AMA is associated with a high probability of antenatal, intrapartum, and obstetric complications. This may reflect the general health status of older mothers as well as their poorer adaptation to increased hemodynamic demands of pregnancy, and thus increased potential health risks for newborns [15,17].

The biological characteristics of the mother had a significant impact on the association between maternal age and birth weight. Model 2 analyzed in the present study showed that a shorter gestational age was strongly associated with a reduced birth weight compared to crude model 1. This result was also confirmed by previous studies [15,37], which indicated that preterm delivery, as well as obstetric complications, were more common among older mothers, and this group had a higher risk of developing pregnancy-induced hypertension and placenta previa. Based on previously published data, it can be concluded that gestational age, marital status, and place of residence may strongly influence birth weight [15,16,23,37]. The results of our study suggest that the factors associated with an increased risk of premature birth are complex, with the demographic background being a significant one connecting the associations between maternal age and decreased birth weight (model 3). Nevertheless, these factors do not negate the evidence that maternal age significantly impacts newborn birth weight. Model 4 included all the previously studied factors as well as the level of education, and this combination did not alter the inverse U-shape, whereas only a minor effect was observed for boys born to primiparous mothers owing to a low number of births recorded for women over the age of 44 years with a lower education level.

The results of this study showed that lower maternal education was associated, at least partly, with inequalities in newborn outcomes. Regardless of parity, the Z-scores reflected the most evident deficits in newborn birth weight after adjusting for the educational factor, and in the case of multiparous women, the values shifted from positive to negative. This may have been due to the lower education level of multiparous women whose primary-to-secondary education ratio was greater compared to primiparous women (58.6% vs. 47.2%). Contrastingly, among multiparous women aged 30–39 years, the Z-scores were not fully consistent with those of the other age groups, which may be due to a higher proportion of births observed among mothers in the age group 30–39 with a higher level of education. The significant influence of differences in maternal educational level on newborn outcomes has also been highlighted in some studies [24,38,39], which indicated that maternal education has a meaningful effect on child health. Mothers with a low level of education may give birth to newborns with a lower weight due to their limited access to specialized health services, delay in the use of antenatal care, or inadequate prenatal monitoring. Furthermore, a low level of education may be insufficient to reassure women of reproductive age about pregnancy planning and proper nutritional habits and is also associated with smoking and psychosocial stress [24]. Inappropriate fetal growth and development can lead to a widened socioeconomic gap in children’s opportunities for mobility in later life, reducing their life chances for welfare and exacerbating social inequalities.

Along with maternal factors liable to inequalities, fetal sex was another significant factor producing potential inequality in newborn outcomes. Compared to girls, the birth weight of boys was higher by 131.0 g among primiparous mothers and 140.9 g among multiparous women. These data are consistent with the findings of other investigators, who demonstrated that the mean difference in birth weight between boys and girls ranged from 111 to 184 g [29,40]. Birth weight differences related to sex could be explained by growth strategies for male and female fetuses, as well as differences in gene expression and the effects of steroids or proteins on placental function [41]. Noticeably, the present study showed that maternal age strongly influenced birth weight in both sexes; however, when considered on a population level, the age of the mother did not influence the difference in birth weight between male and female neonates. This observation indicates that maternal age plays an important—but secondary—role in the determination of birth weight, compared to the sex of the newborn.

### Strengths and Limitations

This study is the first to report the results of birth weight using Z-scores in five-year maternal age groups encompassing parity. Only a few published reports have shown changes in birth weight using Z-scores concerning mothers’ age in a national sample but without division based on parity [42]. Moreover, the strength of this population-based study is that it was performed on a large dataset of 3.8 million live births, which included 263,492 cases of AMA, covering a longer period (between 2008 and 2017) and is an important source of evidence relevant to population health assessment [43]. The data for this study came from a population with a high degree of ethnic homogeneity (99.9% Caucasian). Due to the use of a homogenous national sample, we eliminated the risk of over- or underestimation of the final results. Additionally, the dataset contained a reasonably small proportion of missing and excluded data (6.62%), which is another strength of the study. We believe that our findings will enable clinicians to identify the optimal age of women to have children, as well as to identify critical maternal ages that may be associated with lower birth weight.

Several limitations should be addressed when interpreting the findings of this study. The results obtained are averaged for the entire neonatal population and should not be interpreted as an individual risk. However, the analysis of the complete live birth dataset revealed a clear trend toward lower birth weight, especially in the case of older and less educated mothers. We could not adjust the findings for smoking and antenatal care, both of which were associated with adverse neonatal outcomes [24,44]. Unfortunately, these data were missing in the birth card registry and were therefore excluded from the analysis. However, some population studies demonstrated that 12.2% of women in Poland reported smoking while pregnant. This proportion varied across different age groups of pregnant women i.e., it was 27.2% in those aged under 23 years and ranged between 10.5% and 11.1% among women aged 23–31 years, whereas it was 9% after 31 years of age. Furthermore, active smoking was reported more often in pregnant women with low education levels, reaching approximately 40% [45]. Despite a large and comprehensive data collection, there was no feasibility to evaluate the associations between birth weight and possible confounders, such as body weight and general health status of pregnant women. Thus, BMI, hypertension, and diabetes, which are usually associated with neonatal outcomes [4,17,46], were not included in this study design, although these risk factors may be of importance. According to some data reported elsewhere, underweight during pregnancy, presumably resulting in inadequate nutrient supply in fetuses, may be found in 9.5% of pregnant women in Poland [47]. Our analysis provided evidence demonstrating inequalities in birth weight related mainly to maternal factors, especially age and education.

## 5. Conclusions

Regardless of parity, advanced maternal age was strongly associated with decreased neonatal birth weight, implying complications in early pregnancy and the antenatal period as well as obstetric complications. The findings of this study may have clinical significance and may be useful in preconception and pregnancy counseling to support women’s family planning decisions, particularly the consequences of maternity delay. The usefulness of the data obtained in this study may confer a key role for public health by providing an opportunity to inform about the health needs of mothers and children. The finding of birth weight inequalities related to maternal education emphasizes the importance of social policies aimed at improving women’s education during their reproductive age to alleviate unfavorable newborn outcomes.

## Figures and Tables

**Table 1:** Maternal and newborn characteristics analyzed in the study for the period between 2008 and 2017.

Variable		Primiparas						Multiparas					
Sex of Newborn		Total *n* = 1,742,641		Boys *n* = 897,868		Girls *n* = 844,773		Total *n* = 1,914,942		Boys *n* = 985,499		Girls *n* = 929,443	
		*n*	%	*n*	%	*n*	%	*n*	%	*n*	%	*n*	%
Birth weight [gram]	<2500	80,304	4.6	37,655	4.2	42,649	5.1	78,745	4.1	36,956	3.7	41,789	4.5
	2500–3999	1,496,129	85.9	749,891	83.5	746,238	88.3	1,593,096	83.2	789,441	80.1	803,655	86.5
	≥4000	166,208	9.5	110,322	12.3	55,886	6.6	243,101	12.7	159,102	16.1	83,999	9.0
Maternal age groups [years]	20–24	491,921	28.2	253,627	28.2	238,294	28.2	186,581	9.7	95,733	9.7	90,848	9.8
	25–29	774,608	44.5	399,341	44.5	375,267	44.4	574,628	30.0	295,568	30.0	279,060	30.0
	30–34	374,259	21.5	192,586	21.4	181,673	21.5	743,303	38.9	383,020	38.9	360,283	38.8
	35–39	89,295	5.1	45,894	5.1	43,401	5.1	342,166	17.9	176,105	17.9	166,061	17.9
	40–44	12,120	0.7	6171	0.7	5949	0.7	65,497	3.4	33,661	3.4	31,836	3.4
	≥45	438	0.0	249	0.0	189	0.0	2767	0.1	1412	0.1	1355	0.1
Gestational age [week]	<37	95,661	5.5	52,576	5.9	43,085	5.1	105,938	5.5	58,624	5.9	47,314	5.1
	37–41	1,609,252	92.3	826,194	92.0	783,058	92.7	1,781,029	93.0	912,737	92.6	868,292	93.4
	≥42	37,728	2.2	19,098	2.1	18,630	2.2	27,975	1.5	14,138	1.4	13,837	1.5
Stillbirth history ^1^		Not applicable						13,439	0.7	6984	0.7	6455	0.7
Marital status	Married	1,285,592	73.8	662,490	73.8	623,102	73.8	1,627,734	85.0	838,120	85.0	789,614	85.0
	Unmarried	457,049	26.2	235,378	26.2	221,671	26.2	287,208	15.0	147,379	15.0	139,829	15.0
Place of residence	Urban	1,071,426	61.5	551,524	61.4	519,902	61.5	1,074,058	56.1	553,125	56.1	520,933	56.0
	Rural	671,215	38.5	346,344	38.6	324,871	38.5	840,884	43.9	432,374	43.9	408,510	44.0
Maternal education	Low	54,718	3.1	27,963	3.1	26,755	3.2	145,153	7.6	74,288	7.5	70,865	7.6
	Medium	769,353	44.1	396,830	44.2	372,523	44.1	977,191	51.0	502,708	51.0	474,483	51.1
	High	918,570	52.7	473,075	52.7	445,495	52.7	792,598	41.4	408,503	41.5	384,095	41.3

^1^ only multiparous mothers were included.

**Table 2:** Distribution of birth weight in relation to maternal age stratified by parity and sex of neonates.

**Maternal Age Groups [Years]**	**Primiparas**				**Multiparas**				**^††^ *p***		
	**Total** ***n* = 1,742,641**	**Boys** ***n* = 897,868**	**Girls** ***n* = 844,773**	**^†^ *p***	**Total** ***n* = 1,914,942**	**Boys** ***n* = 985,499**	**Girls** ***n* = 929,443**	**^†^ *p***	**Total**	**Boys**	**Girls**
	Mean birth weight [grams] ± SD										
20–24	3362.1 ± 520.6	3425.3 ± 532.6	3294.9 ± 498.7	***	3366.6 ± 536.4	3437.4 ± 545.7	3292.0 ± 516.1	***	**	***	NS
25–29	3373.1 ± 513.6	3436.7 ± 523.4	3305.5 ± 494.0	***	3438.3 ± 523.0	3509.3 ± 531.3	3363.1 ± 503.3	***	***	***	***
30–34	3337.5 ± 535.4	3400.6 ± 545.5	3270.6 ± 516.1	***	3441.5 ± 528.3	3509.3 ± 538.8	3369.4 ± 507.1	***	***	***	***
35–39	3279.3 ± 572.4	3343.9 ± 584.8	3211.0 ± 550.8	***	3402.8 ± 565.7	3468.2 ± 576.9	3333.4 ± 545.0	***	***	***	***
40–44	3199.2 ± 617.5	3268.4 ± 636.3	3127.5 ± 588.9	***	3343.1 ± 617.2	3403.2 ± 634.5	3279.5 ± 591.8	***	***	***	***
≥45	3106.9 ± 627.0	3207.7 ± 633.8	2974.0 ± 594.0	***	3258.3 ± 663.8	3329.6 ± 673.4	3184.0 ± 645.5	***	***	**	**
Total	3356.3 ± 524.9	3419.8 ± 535.6	3288.8 ± 504.6	***	3422.7 ± 538.6	3491.1 ± 548.8	3350.2 ± 517.9	***	***	***	***
	Mean Z-score ± SD										
20–24	−0.11 ± 0.98	−0.11 ± 0.98	−0.10 ± 0.98	***	−0.02 ± 1.00	−0.01 ± 1.00	−0.03 ± 1.01	***	***	***	***
25–29	−0.05 ± 0.95	−0.06 ± 0.96	−0.05 ± 0.95	***	0.13 ± 0.99	0.14 ± 0.99	0.12 ± 1.00	***	***	***	***
30–34	−0.06 ± 0.96	−0.07 ± 0.96	−0.06 ± 0.96	***	0.18 ± 0.99	0.18 ± 0.99	0.18 ± 0.99	**	***	***	***
35–39	−0.08 ± 0.98	−0.08 ± 0.98	−0.08 ± 0.98	NS	0.17 ± 1.02	0.17 ± 1.02	0.17 ± 1.03	NS	***	***	***
40–44	−0.10 ± 1.00	−0.09 ± 1.02	−0.11 ± 0.97	NS	0.12 ± 1.08	0.12 ± 1.09	0.13 ± 1.08	NS	***	***	***
≥45	−0.19 ± 1.06	−0.15 ± 1.12	−0.24 ± 0.98	NS	0.03 ± 1.10	0.03 ± 1.10	0.02 ± 1.10	NS	***	*	***
Total	−0.07 ± 0.96	−0.08 ± 0.96	−0.07 ± 0.96	***	0.14 ± 1.00	0.15 ± 1.00	0.14 ± 1.01	***	***	***	***

^†^ *p*-values for differences in birth weight between boys and girls; ^††^ *p*-values for differences in birth weight between primiparity and multiparity; *** *p* ≤ 0.001; ** *p* ≤ 0.01; * *p* ≤ 0.05; NS—not significant; SD—standard deviation.

**Table 3:** Effects of maternal age (β) in regression models for birth weight stratified by parity and sex of neonates.

Age Groups [Years]	Primiparas				Multiparas			
	Boys		Girls		Boys		Girls	
	β with 95% CI [Grams]	^†^ *p*	β with 95% CI [Grams]	^†^ *p*	β with 95% CI [Grams]	^†^ *p*	β with 95% CI [Grams]	^†^ *p*
Model 1 (crude): maternal age								
20–24	−11.4 (−14.1, −8.8)	***	−10.6 (−13.2, −8.0)	***	−71.9 (−75.9, −67.9)	***	−71.1 (−74.9, −67.2)	***
25–29	Reference		Reference		Reference		Reference	
30–34	−36.1 (−39.0, −33.2)	***	−34.9 (−37.8, −32.1)	***	−0.1 (−2.7, 2.6)	NS	6.3 (3.7, 8.9)	***
35–39	−92.8 (−97.9, −87.6)	***	−94.5 (−99.5, −89.5)	***	−41.1 (−44.4, −37.9)	***	−29.7 (−32.9, −26.6)	***
40–44	−168.3 (−181.8, −154.9)	***	−178.0 (−190.9, −165.4)	***	−106.2 (−112.3, −100.0)	***	−83.6 (−89.6, −77.6)	***
≥45	−229.0 (−295.5, −162.5)	***	−331.5 (−403.3, −259.6)	***	−179.7 (−208.3, −151.0)	***	−179.1 (−206.7, −151.5)	***
^††^ *p*	***		***		***		***	
^†††^ *p*	**		***		***		***	
Model 2: maternal age adjusted for biological factors								
20–24	−13.3 (−15.6, −10.9)	***	−13.3 (−15.6, −11.0)	***	−60.6 (−64.1, −57.0)	***	−59.4 (−62.9, −55.9)	***
25–29	Reference		Reference		Reference		Reference	
30–34	−20.8 (−23.4, −18.3)	***	−21.1 (−23.6, −18.6)	***	7.6 (5.3, 10.0)	***	11.6 (9.3, 13.9)	***
35–39	−52.8 (−57.4, −48.2)	***	−59.4 (−63.9, −54.9)	***	−13.8 (−16.7, −10.9)	***	−7.0 (−9.9, −4.2)	***
40–44	−100.4 (−112.3, −88.6)	***	−111.0 (−122.5, −99.6)	***	−51.9 (−57.4, −46.5)	**	−35.8 (−41.2, −30.4)	***
≥45	−139.6 (−198.1, −81.0)	***	−200.1 (−264.0, −136.1)	***	−109.7 (−135.1, −84.4)	***	−109.8 (−134.6, −85.1)	***
^††^ *p*	***		***		***		***	
^†††^ *p*	*		***		***		***	
Model 3: maternal age adjusted for biological and demographic factors								
20–24	−7.1 (−9.5, −4.7)	***	−7.0 (−9.3, −4.6)	***	−44.3 (−47.8, −40.7)	***	−44.0 (−47.5, −40.5)	***
25–29	Reference		Reference		Reference		Reference	
30–34	−18.5 (−21.1, −15.9)	***	−18.9 (−21.4, −16.4)	***	6.4 (4.1, 8.7)	***	10.3 (8.0, 12.6)	***
35–39	−47.0 (−51.5, −42.4)	***	−53.8 (−58.3, −49.4)	***	−10.8 (−13.7, −8.0)	***	−4.3 (−7.1, −1.4)	**
40–44	−93.0 (−104.8, −81.1)	***	−104.1 (−115.6, −92.6)	***	−45.0 (−50.5, −39.6)	***	−29.8 (−35.2, −24.4)	***
≥45	−130.0 (−188.5, −71.5)	***	−192.7 (−256.6, −128.7)	***	−103.2 (−128.5, −77.9)	***	−105.6 (−130.3, −80.9)	***
^††^ *p*	***		***		***		***	
^†††^ *p*	*		***		***		***	
Model 4: maternal age adjusted for biological, demographic, and social factors								
20–24	5.3 (2.7, 7.9)	***	4.9 (2.4, 7.4)	***	−16.5 (−20.1, −12.9)	***	−18.0 (−21.6, −14.5)	***
25–29	Reference		Reference		Reference		Reference	
30–34	−20.1 (−22.7, −17.6)	***	−20.5 (−23.0, −18.0)	***	−2.0 (−4.4, 0.3)	NS	2.1 (−0.2, 4.4)	NS
35–39	−47.7 (−52.3, −43.2)	***	−54.6 (−59.1, −50.2)	***	−16.5 (−19.4, −13.7)	***	−9.9 (−12.7, −7.1)	***
40–44	−92.3 (−104.2, −80.5)	***	−103.5 (−115.0, −92.1)	***	−44.3 (−49.7, −38.8)	***	−29.6 (−35.0, −24.3)	***
≥45	−131.0 (−189.4, −72.6)	***	−192.3 (−256.1, −128.5)	***	−99.4 (−124.6, −74.3)	***	−101.9 (−126.6, −77.3)	***
^††^ *p*	***		***		***		***	
^†††^ *p*	NS		***		***		***	

^†^ *p*-value for given maternal age category versus reference category; ^††^ *p*-value for linear term; ^†††^ *p*-value for quadratic term; CI—confidence interval; *** *p* ≤ 0.001; ** *p* ≤ 0.01; * *p* ≤ 0.05; NS—not significant.

**Table 4:** Birth weight Z-scores estimated in relation to maternal age groups, stratified by parity and sex of neonates.

Age Group [Years]	Primiparas		Multiparas	
	Boys	Girls	Boys	Girls
	Estimated Birth Weight with 95% CI [Z-Scores]			
Model 1 (crude): maternal age				
20–24	−0.110 (−0.114, −0.106)	−0.101 (−0.105, −0.097)	−0.008 (−0.014, −0.001)	−0.030 (−0.037, −0.024)
25–29	−0.058 (−0.061, −0.055)	−0.046 (−0.049, −0.042)	0.138 (0.134, 0.141)	0.121 (0.118, 0.125)
30–34	−0.071 (−0.076, −0.067)	−0.058 (−0.062, −0.053)	0.183 (0.180, 0.186)	0.176 (0.173, 0.180)
35–39	−0.081 (−0.090, −0.072)	−0.083 (−0.092, −0.074)	0.170 (0.165, 0.175)	0.171 (0.166, 0.176)
40–44	−0.093 (−0.118, −0.069)	−0.114 (−0.138, −0.089)	0.116 (0.106, 0.127)	0.130 (0.119, 0.141)
≥45	−0.150 (−0.270, −0.030)	−0.236 (−0.373, −0.099)	0.032 (−0.021, 0.084)	0.021 (−0.033, 0.074)
Model 2: maternal age adjusted for biological factors				
20–24	−0.068 (−0.074, −0.062)	−0.056 (−0.062, −0.050)	−0.056 (−0.070, 0.041)	−0.067 (−0.082, −0.052)
25–29	−0.016 (−0.022, −0.010)	−0.0003 (−0.006, 0.006)	0.090 (0.077, 0.104)	0.086 (0.072, 0.100)
30–34	−0.030 (−0.037, −0.023)	−0.013 (−0.020, −0.006)	0.135 (0.122, 0.148)	0.140 (0.126, 0.154)
35–39	−0.042 (−0.052, −0.032)	−0.040 (−0.050, −0.029)	0.121 (0.107, 0.134)	0.133 (0.119, 0.148)
40–44	−0.056 (−0.080, −0.031)	−0.074 (−0.099, −0.049)	0.066 (0.049, 0.083)	0.091 (0.073, 0.108)
≥45	−0.114 (−0.234, 0.006)	−0.201 (−0.339, −0.064)	−0.019 (−0.073. 0.035)	−0.020 (−0.075, 0.035)
Model 3: maternal age adjusted for biological and demographic factors				
20–24	−0.073 (−0.080, −0.067)	−0.062 (−0.068, −0.055)	−0.091 (−0.105, −0.077)	−0.100 (−0.115, −0.085)
25–29	−0.035 (−0.041, −0.029)	−0.022 (−0.028, −0.015)	0.018 (0.005, 0.032)	0.017 (0.003, 0.031)
30–34	−0.045 (−0.051, −0.038)	−0.030 (−0.037, −0.023)	0.060 (0.046, 0.073)	0.068 (0.054, 0.082)
35–39	−0.049 (−0.059, −0.038)	−0.049 (−0.059, −0.038)	0.055 (0.041, 0.069)	0.070 (0.056, 0.084)
40–44	−0.059 (−0.084, −0.035)	−0.079 (−0.104, −0.054)	0.009 (−0.008, 0.026)	0.035 (0.018, 0.053)
≥45	−0.113 (−0.232, 0.007)	−0.206 (−0.343, −0.068)	−0.077 (−0.130, −0.023)	−0.079 (−0.134, −0.024)
Model 4: maternal age adjusted for biological, demographic, and social factors				
20–24	−0.119 (−0.126, −0.112)	−0.112 (−0.119, −0.104)	−0.066 (−0.080, −0.051)	−0.073 (−0.088, −0.059)
25–29	−0.110 (−0.117, −0.103)	−0.100 (−0.107, −0.093)	−0.022 (−0.036, −0.009)	−0.021 (−0.035, −0.007)
30–34	−0.123 (−0.131, −0.115)	−0.112 (−0.120, −0.104)	−0.002 (−0.016, 0.011)	0.008 (−0.006, 0.022)
35–39	−0.125 (−0.136, −0.114)	−0.129 (−0.140, −0.118)	−0.0004 (−0.014, 0.014)	0.017 (0.003, 0.032)
40–44	−0.132 (−0.157, −0.108)	−0.156 (−0.182, −0.131)	−0.030 (−0.047, −0.013)	−0.002 (−0.020. 0.015)
≥45	−0.189 (−0.308, −0.069)	−0.283 (−0.420, −0.146)	−0.108 (−0.161, −0.055)	−0.107 (−0.162, −0.052)

## Data Availability

Data were collected from public datasets analyzed or generated during the study and presented in Table 1.
